# DNA methylation-mediated suppression of TUSC1 expression regulates the malignant progression of esophagogastric junction cancer

**DOI:** 10.1186/s13148-024-01689-9

**Published:** 2024-07-23

**Authors:** Zhiqiang Liu, Ganshu Xia, Xiaolong Liang, Baozhong Li, Jingyu Deng

**Affiliations:** 1 Department of Gastric Surgery, Tianjin Medical University Cancer Institute and Hospital, National Clinical Research Center for CancerKey Laboratory of Cancer Prevention and Therapy, TianjinTianjin’s Clinical Research Center for Cancer, Tianjin, 300060 China; 2 Department of Gastric Surgery, Anyang Tumor Hospital, Anyang, 455000 China

**Keywords:** TUSC1, DNA methylation, Esophagogastric junction cancer, Malignant progression

## Abstract

**Background:**

Esophagogastric junction cancer (EJC) refers to malignant tumors that develop at the junction between the stomach and the esophagus. TUSC1 is a recently identified tumor suppressor gene known for its involvement in various types of cancer. The objective of this investigation was to elucidate the regulatory influence of DNA methylation on TUSC1 expression and its role in the progression of EJC.

**Methods:**

Bioinformatics software was utilized to analyze the expression of TUSC1, enriched pathways, and highly methylated sites in the promoter region. TUSC1 expression in EJC was assessed using quantitative reverse transcription polymerase chain reaction (qRT-PCR), western blot (WB), and immunohistochemistry. Methylation-specific PCR was employed to detect the methylation level of TUSC1. To analyze the effects of TUSC1 and 5-AZA-2 on tumor cell proliferation, migration, invasion, cell cycle, and apoptosis, several assays including CCK-8, colony formation, transwell, and flow cytometry were conducted. The expression of MDM2 was assessed using qRT-PCR and WB. WB detected the expression of p53, and p-p53, markers for EJC cell proliferation, epithelial-mesenchymal transition, and apoptosis. The role of TUSC1 in tumor occurrence in vivo was examined using a xenograft mouse model.

**Results:**

TUSC1 expression was significantly downregulated in EJC. Overexpression of TUSC1 and treatment with 5-AZA-2 inhibited the malignant progression of EJC cells. In EJC, low methylation levels promoted the expression of TUSC1. Upregulation of TUSC1 suppressed the expression of MDM2 and activated the p53 signaling pathway. Inactivation of this pathway attenuated the inhibitory effect of TUSC1 overexpression on EJC cell proliferation, migration, invasion, and other behaviors. Animal experiments demonstrated that TUSC1 overexpression inhibited EJC tumor growth and metastasis in vivo.

**Conclusion:**

TUSC1 was commonly downregulated in EJC and regulated by methylation. It repressed the malignant progression of EJC tumors by mediating the p53 pathway, suggesting its potential as a diagnostic and therapeutic target for EJC.

**Supplementary Information:**

The online version contains supplementary material available at 10.1186/s13148-024-01689-9.

## Introduction

Esophagogastric junction cancer (EJC), primarily adenocarcinoma of the esophageal-gastric junction (AEG), is characterized by tumors located within a 5 cm range above and below the junction of the esophagus and stomach. Several risk factors, such as obesity, gastroesophageal reflux disease, and Barrett esophagus, have been associated with AEG [1]. While the global incidence of gastric cancer (GC) has declined in recent decades, that of EJC has increased [2]. To improve survival rates, multimodal treatments involving surgery, chemotherapy, and radiation therapy have been developed and implemented for EJC patients [3]. However, given the nonspecific clinical symptoms in the early stages of EJC, most patients present with advanced disease at diagnosis that precluded surgical intervention. Although radiotherapy and chemotherapy have extended the survival time for locally advanced EJC patients, the prognosis for late-stage EJC remains generally unfavorable, with a grim 5-year overall survival rate [2]. Therefore, a deeper understanding of the etiology and molecular mechanisms underlying EJC is crucial for the development of innovative diagnostic methods, treatment strategies, and preventive approaches.

DNA methylation is a process in which DNA methyltransferases reversibly add a methyl group from S-adenosylmethionine to the fifth carbon atom of the cytosine in DNA sequences, forming 5-methylcytosine [4]. DNA methylation was the first epigenetic modification shown to be closely associated with tumorigenesis, providing a stable mechanism for gene silencing [5]. Aberrant DNA methylation has been extensively studied and found to disrupt the typical expression and functionality of numerous genes implicated in tumor regulation, thereby influencing both the initiation and progression of tumorigenesis [6]. DNMT3b accelerates promoter methylation of FLI1, resulting in the downregulation of FLI1 expression, which enhances proliferation, migration, and invasion of colorectal cancer (CRC) cells [7]. Reports have also documented the influence of DNA methylation alterations on the advancement of gastric and esophageal cancer. For instance, Teng et al. [5] found that low expression of PRDM5 is associated with promoter methylation, and its downregulation contributes to tumor proliferation and migration, correlating with poor prognosis in GC. Long noncoding RNA ADAMTS9-AS2 is downregulated in esophageal cancer, and research has shown that ADAMTS9-AS2 can repress the proliferation, invasion, and migration of esophageal cancer by mediating CDH3 promoter methylation [8]. Nevertheless, the role of DNA methylation in EJC has been rarely explored, and further investigation into the molecular mechanisms of DNA methylation in the EJC cancer gene may hold significant potential for future cancer therapies.

Tumor initiation and progression represent a complex pathological process involving multiple factors, stages, and genetic alterations, where the loss of tumor suppressor gene function and activation of oncogenes serve as the molecular basis for cellular carcinogenesis [9]. For instance, circ_PLXNB1 is downregulated in CRC and can suppress CRC progression by upregulating tumor suppressor candidate 1 (TUSC1) through binding to miR-4701-5p [10]. TUSC1 is a recently identified tumor suppressor gene that is frequently inactivated through gene methylation in various human malignancies, playing a crucial role in tumorigenesis [11]. For instance, in GC, decreased TUSC1 mRNA expression is implicated in poor prognosis of patients, and TUSC1 is typically suppressed in cancer cells due to high levels of gene methylation [12]. However, promoter methylation of the TUSC1 gene in EJC has rarely been reported. In addition, previous studies lacked sufficient functional analysis of TUSC1, and the mechanism of TUSC1 promoter methylation in EJC affecting tumor progression has not been elucidated. Therefore, our study further explored the molecular mechanisms by which TUSC1 promoter methylation affects EJC cells.

To investigate the role of TUSC1 in EJC, this study examined the expression and methylation levels of TUSC1 and probed into the impact of 5-AZA-2-mediated demethylation and TUSC1 overexpression on cancer cell behavior. Bioinformatics, clinical analysis, and cell experiments confirmed that TUSC1 was downregulated in EJC. In vitro experiments identified methylation sites in the promoter region of TUSC1 and demonstrated their regulatory role in TUSC1 expression. Additionally, it was determined that TUSC1 influences the malignant progression of EJC cells through the p53 signaling pathway. Animal experiments further supported the findings, showing that aberrant TUSC1 expression affected EJC tumor growth and metastasis. In summary, abnormal DNA methylation of TUSC1 mediated the critical role of the p53 signaling pathway in EJC tumorigenesis, suggesting the potential of TUSC1 as a diagnostic and prognostic marker for predicting the survival of individuals with EJC.

## Materials and methods

### Clinical samples

Between June 2022 and July 2023, a total of 56 pairs of EJC tissues, adjacent tissues (2–3 cm away from EJC tissues), and normal tissues (at least 5 cm away from EJC tissues) were collected from individuals who underwent surgical resection at Anyang Cancer Hospital. All patients were newly diagnosed, had complete clinical data, and did not have any other malignancies. Individuals who had received preoperative radiotherapy or chemotherapy were excluded from the study. The research was approved by the ethics committee of Anyang Cancer Hospital, and informed consent was obtained from all patients.

### Bioinformatics analysis

The Shiny Methylation Analysis Resource Tool (SMART), a web application for comprehensive analysis of DNA methylation data from the Cancer Genome Atlas (TCGA) project, was used. Data were obtained from the UCSC Xena public data center. To examine the molecular mechanisms contributing to the irregular TUSC1 expression in EJC, we employed the MethPrimer online database to investigate the association between TUSC1 mRNA expression and DNA methylation. EJC was selected in MethPrimer, and an analysis of CpG sites in the TUSC1 gene was performed. Gene Expression Profiling Interactive Analysis (GEPIA), an interactive web server for analyzing RNA sequencing expression data from TCGA and the Genotype-Tissue Expression (GTEx) project, was utilized to analyze RNA sequencing expression data from tumor and normal samples. Finally, Gene Set Enrichment Analysis (GSEA) software was utilized for pathway enrichment analysis of the target gene mRNA.

### Transcriptome sequencing

RNA samples were meticulously evaluated for their purity, concentration, and structural integrity utilizing molecular biology instrumentation. This assessment aimed to guarantee the utilization of high-quality samples for subsequent transcriptome sequencing. After the sample assessment, library construction was performed. After that, initial quantification was performed. The library fragment distribution was assessed using the Agilent 2100 DNA 1000 kit after library dilution. Following this, qRT-PCR was employed to precisely determine the library’s effective concentration, ensuring that it exceeded 2 nM and met the requisite quality standards. Qualified libraries were pooled as required and sequenced using the Illumina platform.

### Cell culture

In the eighth edition of the American Joint Committee on Cancer (AJCC6) staging system, malignancies involving the junction of the stomach and esophagus are traditionally considered GC. Since there are no specific cell lines cultured for EJC, and considering the similarity in pathological types, GC cell lines were chosen as alternative experimental models. In this set of experiments, three human GC cell lines, HGC-27, AGS, and NCI-N87, purchased from Wuhan Procell Life Science & Technology Co., Ltd. (China), were selected as candidate cell lines, and the human normal gastric epithelial cell line GES-1 (SUNNCELL, Wuhan, China) was used as a negative control. AGS, NCI-N87, and GES-1 cells were cultured in the RPMI-1640 medium, while HGC-27 cells were cultured in a high-glucose DMEM medium. The culture media were supplemented with 10% fetal bovine serum and 1% penicillin/streptomycin, and all cells were maintained at 37 °C in a 5% CO_2_ [13].

### Cell transfection

The oe-TUSC1 plasmid, oe-MDM2 plasmid, and control (oe-NC) plasmid were synthesized by Fenghui Biotechnology (China) and transfected into cells using UltraFection 3.0 (4A BIOTECH, China) according to the provided protocol. AGS cells were treated with 20 μM p53 signaling pathway inhibitor pifithrin-α (PFT-α) (MCE, USA) [14].

Cells were initially seeded at a density of 1 × 10^6^ cells/ml. After a 24-h incubation period, cells were treated with a 10 μM DNA methyltransferase inhibitor, specifically 5-AZA-2 (MCE, USA), along with an equivalent concentration of DMSO for 48 hours [15].

### Quantitative reverse transcription polymerase chain reaction (qRT-PCR)

Total RNA was isolated from cells utilizing the TRIzol method (Cwbio, China). RNA concentration and purity were determined using a microplate spectrophotometer (OD260/OD280 and OD260/OD230). 2 μg of total RNA was reverse transcribed to synthesize cDNA utilizing the Hifair® II 1st Strand cDNA Synthesis Kit (Yeasn, China). The qRT-PCR analysis of gene expression was performed using UltraSYBR Mixture (Cwbio, China), and the results were quantified and normalized utilizing the 2^−ΔΔCt^ method. GAPDH was used as the reference gene. Primer sequences are provided in Table 1.

**Table 1:** The qRT-PCR primer sequences

Gene	Primer sequence (5’ → 3’)
TUSC1	F: TCTACAGGAACCCGACTCC
	R: GAAGGCACCGTAGTCCAAG
MDM2	F: TGGGCAGCTTGAAGCAGTTG
	R: CAGGCTGCCATGTGACCTAAGA
Ki67	F: GCACCTGCTTGTTTGGAAG
	R: TTGTGTTGGATTTGTGGAACTG
Caspase-3	F: GGGATCGTTGTAGAAGTCTAACTG
	R: CGGCCTCCACTGGTATTTTATG
E-cadherin	F: CCCAATACATCTCCCTTCACAG
	R: CCACCTCTAAGGCCATCTTTG
Vimentin	F: CGTGAATACCAAGACCTGCTC
	R: GGAAAAGTTTGGAAGAGGCAG
GAPDH	F: ACATCGCTCAGACACCATG
	R: TGTAGTTGAGGTCAATGAAGGG

### Western blot (WB)

Total proteins were extracted from cells or tissues utilizing a protein extraction reagent kit. Protein concentration was quantified by utilizing the BCA assay kit (Solarbio, China). Approximately 20 μg of protein extracts was isolated by 6% or 10% SDS-PAGE gel electrophoresis and transferred to PVDF membranes. The membranes were blocked and then kept overnight at 4 °C with primary antibodies against TUSC1, Ki67, caspase-3, E-cadherin, vimentin, mTOR, p-mTOR, p53, and p-p53 (ABclonal, China). After washing, the membranes were incubated with horseradish peroxidase-conjugated secondary antibody (IgG) (Beyotime, China) at room temperature for 1 h. Protein bands were visualized utilizing the Super-sensitive ECL chemiluminescence reagent (Beyotime, China).

### CCK-8 assay

Cells from each group were seeded in 96-well plates (2000 cells/well) with 3 replicate wells per group. After incubation for 0, 24, 48, and 72 h, 10 μL of CCK-8 solution was added to each well, and cells were incubated in the dark for 2 h. The optical density values at 450 nm were read using an enzyme-linked immunosorbent assay (ELISA) reader. The average value of three wells from each group was calculated to generate a cell growth curve.

### Methylation-specific PCR (MSP)

Genomic DNA was extracted from cells, and modified genomic DNA was prepared following the instructions of a methylation-specific PCR kit (Tiangen Biotech, Beijing, China). Modified genomic DNA was used as a template for PCR amplification. The MSP primers were designed using MethPrimer online software, which is provided in Table 2.

**Table 2:** The MSP primer sequences

TUSC1	Primer sequence (5’ → 3’)
Methylation	F: GTAGTGTTTAGGTTTGTACGGAAGC
	R: TAACTTAAAACCAAAAAAAACCGAA
Unmethylation	F: AGTGTTTAGGTTTGTATGGAAGTGT
	R: TAACTTAAAACCAAAAAAAACCAAA

The PCR amplification system (25 µl) consisted of 2.5 µl of 10 × buffer, 1.0 µl of dNTP, 1 µl of each methylated or unmethylated primer, 2 µl of DNA template, and 2 µl of MgCl_2_, with dH2O added to a total volume of 25 µl. The PCR parameters included an initial denaturation step at 95 °C for 5 min, followed by denaturation at 95 °C for 30 s, annealing at 30 s, and extension at 72 °C for 30 s. The PCR products were then electrophoresed on a 2% agarose gel, and images were scanned using a UV gel imaging system.

### 5-AZA-2 treatment of cells

The cells were subjected to treatment with varying concentrations of 5-AZA-2, specifically 0.1 μM, 10 μM, and 100 μM, over 48 h. The collected cells were further analyzed. Simultaneously, EJC cells were cultured without drug treatment as a control [16].

### Immunohistochemistry (IHC)

Patient or mouse tumor tissue specimens were fixed, embedded in paraffin, and prepared into tissue sections. The 5-μm-thick tissue sections were deparaffinized, dehydrated, and subjected to heat-induced antigen retrieval in EDTA antigen retrieval solution for 20 min. The tissue sections were incubated overnight at 4 °C with primary antibodies against TUSC1, Ki67, caspase-3, E-cadherin, and vimentin (ABclonal, China), followed by incubation with secondary antibody for an additional 2 h. DAB staining was performed on the sections using the DAB reagent kit (ZSGB-Bio, Beijing, China).

### Colony formation assay

To form colonies, treated EJC cells were seeded at a density of 400 cells per well in a 12-well plate. After two weeks, the cells were fixed with 75% ethanol and stained with 5% (w/w) crystal violet for 30 min. The excess staining solution was washed away slowly with water, and the number of cell colonies was observed.

### Transwell assay

Transfected EJC cells (1 × 10^4^ cells/well) were seeded in transwell chambers (Corning, USA) for migration assay or in chambers pre-coated with 100 μL of Matrigel matrix gel (BD Biosciences, USA) for invasion assay. Serum-free medium and complete medium were added to the upper and lower chambers, respectively. After incubation for 24 h (for migration) or 72 h (for invasion), the invading cells were fixed with 75% ethanol, stained with 5% crystal violet, and photographed under a microscope.

### Flow cytometry

For apoptosis detection, post-treated EJC cells were collected, washed with PBS, and then suspended in 500 μL of 1 × binding buffer, with a cell concentration of 1 × 10^6^ cells/mL. V-FITC-conjugated annexin V (5 µL) and propidium iodide (PI, 10 μL) were introduced to the 500 μL cell suspension. The mixture was then incubated for 15 min at room temperature, shielded from light. Flow cytometry was used for detection.

To assess the cell cycle, transfected cells were harvested and washed with PBS. Cells were fixed with 75% cold ethanol dropwise and incubated at 4 °C for 2 h. After centrifugation at 1500 rpm for 5 min and two washes with PBS, cells were resuspended in 500 μL of PI staining solution (BD, USA) at room temperature, avoiding light, and incubated for 10 min. Flow cytometry was employed for cell cycle analysis.

### Nude mouse model establishment

Female BALB/c nude mice (4–5 weeks old, weighing 16–20 g) were obtained from Shanghai SLAC Laboratory Animal Co., Ltd. (China) and housed in a SPF-grade animal facility. Ethical approval for the animal experiments was obtained from the Ethics Committee of Anyang Tumor Hospital. Six female BALB/c nude mice were put into two groups (n = 3 per group). Stable transfected human EJC cell lines with oe-NC and oe-TUSC1 were subcutaneously implanted on the right side of the nude mice. Tumor formation was observed for 4 weeks, followed by euthanasia of the nude mice. The transplanted tumors were dissected, harvested, and weighed. Tumor volume (V) was calculated using the following formula: V (mm^3^) = length (mm) × width (mm)^2^/2. Expression of TUSC1, proliferation, epithelial-mesenchymal transition (EMT), and apoptosis marker proteins in the tumor tissues of each group of nude mice was assayed utilizing qRT-PCR, WB, and IHC.

### Statistical analysis

All experiments were repeated at least three times independently, and all data are presented as mean ± standard deviation (SD). Statistical analysis was performed using GraphPad Prism 8.0 software. Group differences were determined using a chi-square test or *t*-test. * represents 0.01 ≤ *P* < 0.05, ** represents 0.001 ≤ *P* < 0.01, *** represents 0.0001 ≤ *P* < 0.001, **** represents *P* < 0.0001, and *P* < 0.05 was considered statistically significant.

## Results

### Expression of TUSC1 in EJC tissues and cells

**Fig. 1:**
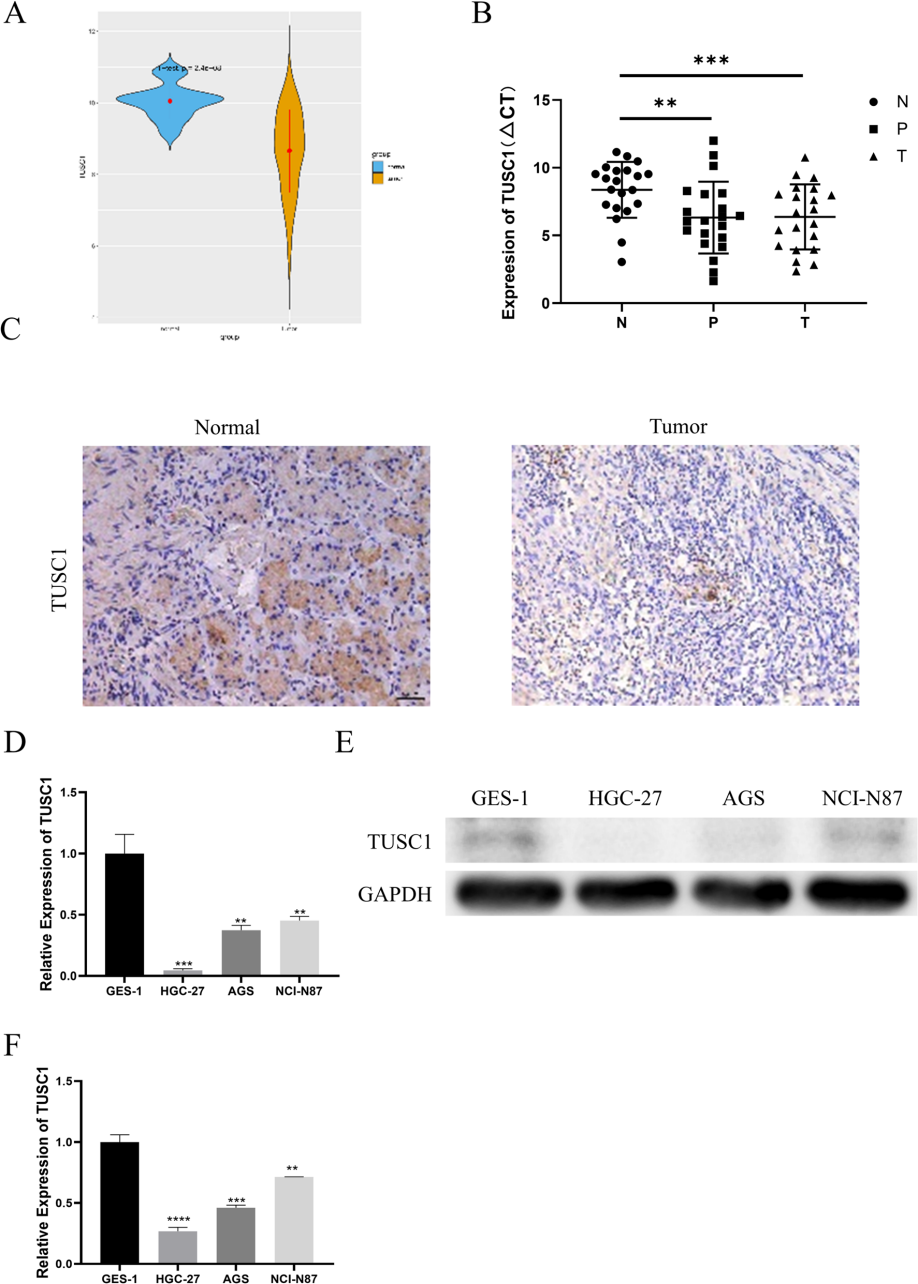
Expression of TUSC1 in EJC tissues and cells. **A** Bioinformatics analysis of TUSC1 expression in EJC patients. **B**–**C** qRT-PCR and IHC analysis of TUSC1 expression in clinical EJC tissues. **D**–**F** qRT-PCR and WB analysis of TUSC1 expression in EJC cells. ** represents 0.001 ≤ *P* < 0.01, *** represents 0.0001 ≤ *P* < 0.001, **** represents *P* < 0.0001

In this study, a total of 56 EJC patients with complete clinical information were recruited, and the clinical pathological information is summarized in Table 3. Bioinformatics analysis of TUSC1 expression in EJC-related databases revealed a significant downregulation of TUSC1 expression in EJC patients compared to normal individuals (Fig. 1A). qRT-PCR was used for detecting the expression of TUSC1 in 21 EJC tissues, adjacent tissues, and normal tissues, while IHC was employed to assess TUSC1 expression in 56 EJC tissues and adjacent tissues. The results showed a significant downregulation of TUSC1 expression in tumor tissues (Fig. 1B–C). Additionally, we examined TUSC1 expression in human normal gastric epithelial cells (GES-1) and EJC cells (HGC-27, AGS, NCI-N87) using qRT-PCR and WB. The findings demonstrated that TUSC1 exhibited a decreased expression in EJC cells compared to the control (Fig. 1D–F). These findings collectively suggested that TUSC1 was downregulated in EJC. Based on the median TUSC1 expression level in the 56 patients, patients with expression levels higher than the median were categorized as TUSC1 high-expression patients (Supplementary Table 1). Subsequent clinical analysis revealed associations between TUSC1 expression and tumor size, T-stage, N-stage, and nerve invasion (Table 4).

**Table 3:** Clinical information

Characteristics	*N*(%)
*Age (years)*	
≤ 65	22(39.29)
> 65	34(60.71)
*Gender*	
Male	44(78.57)
Female	12(21.43)
*Tumor size (cm)*	
≤ 4	37(66.07)
> 4	19(33.93)
*T-stage*	
I–II	30(53.57)
III-IV	26(46.43)
*N-stage*	
N0-1	42(75.00)
N2–3	14(25.00)
*Nerve invasion*	
Negative	34(60.71)
Positive	22(39.29)

**Table 4:** Correlation analysis of TUSC1 expression and clinicopathological features

Characteristics	n	TUSC1 high-expression N (%)	TUSC1 low-expression N (%)	*P*-value
*Age (years)*				
≤ 65	22	11(50.00)	11(50.00)	> 0.05
> 65	34	17(50.00)	17(50.00)	
*Gender*				
Male	44	21(47.73)	23(52.27)	> 0.05
Female	12	7(58.33)	5(41.67)	
*Tumor size (cm)*				
≤ 4	37	22(59.46)	15 (40.54)	0.0482
> 4	19	6(31.58)	13(68.42)	
*T-stage*				
I–II	30	19(63.33)	11(36.67)	0.0321
III-IV	26	9(34.62)	17(65.38)	
*N-stage*				
N0-1	42	26(61.90)	16(38.10)	0.0020
N2–3	14	2(14.29)	12(85.71)	
*Nerve invasion*				
Negative	34	21(61.76)	13(38.24)	0.0286
Positive	22	7(31.82)	15(68.18)	

### DNA methylation in the TUSC1 promoter region modulates TUSC1 expression

**Fig. 2:**
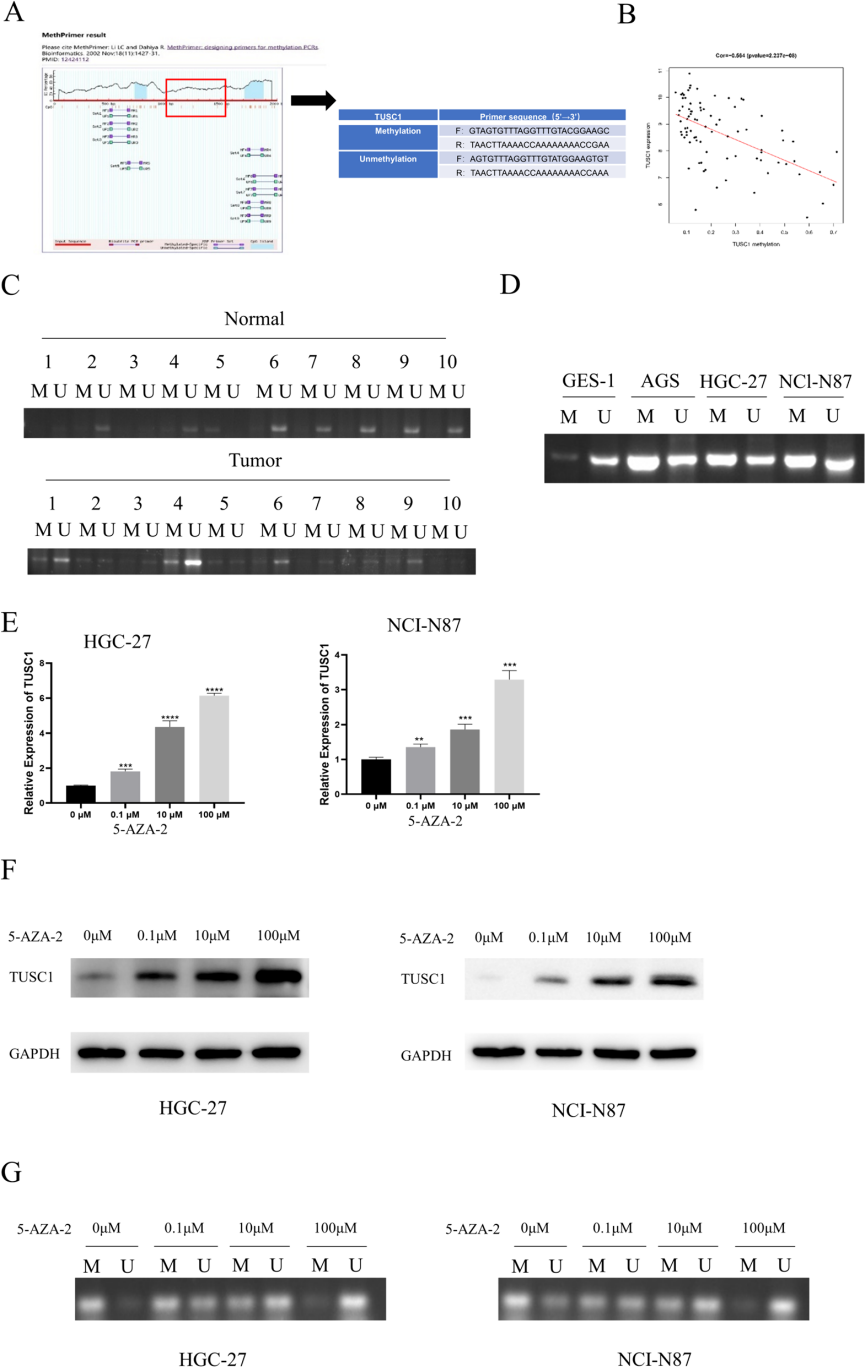

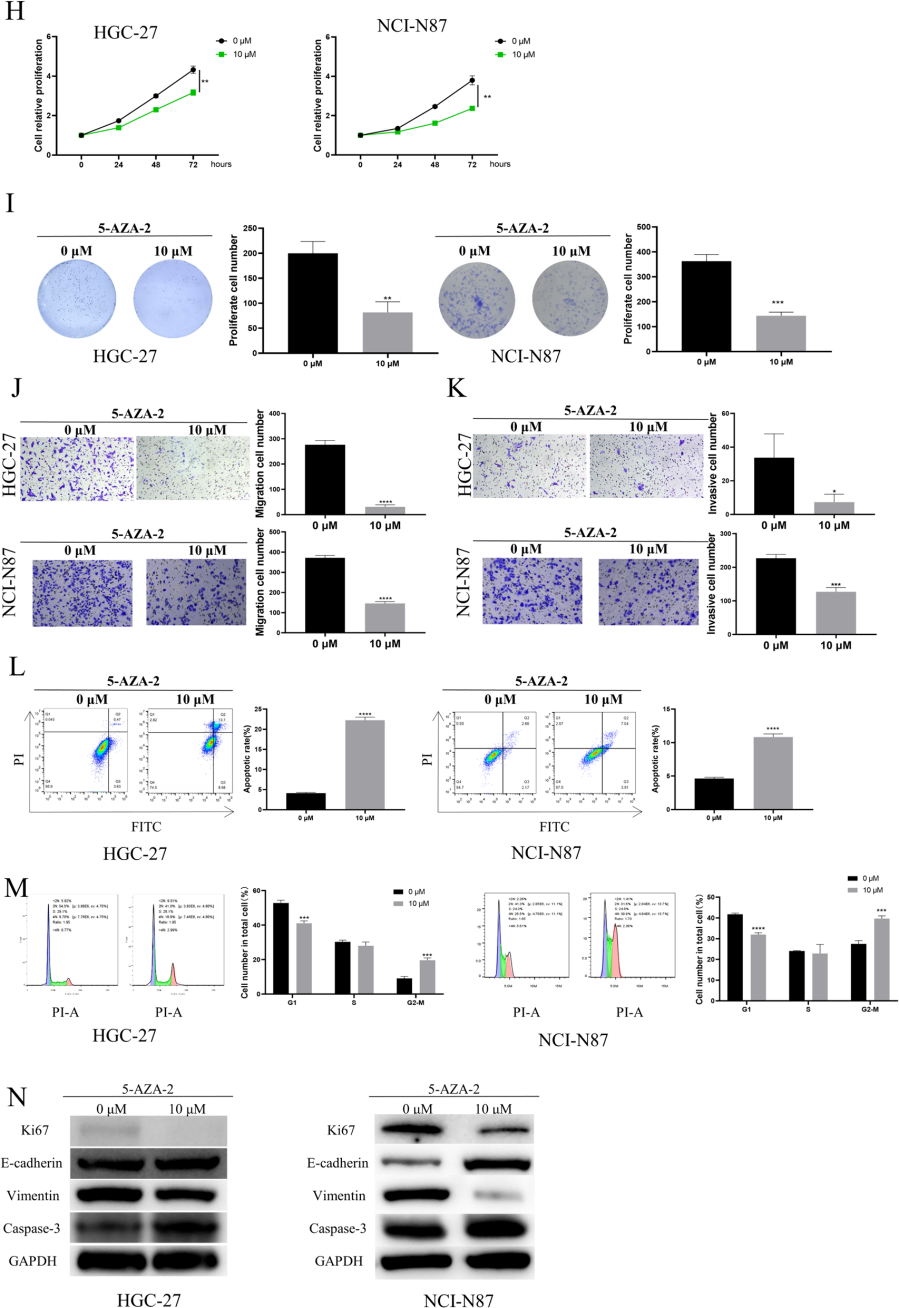
Identification of DNA methylation in the TUSC1 promoter region in EJC cells. **A** Schematic representation of CpG islands within the TUSC1 promoter, as determined by bioinformatics analysis; TUSC1 promoter methylation site map. **B** Relationship between TUSC1 expression levels and DNA methylation extent as revealed by bioinformatics analysis. **C** Methylation levels of TUSC1 in normal tissues compared to EJC tissues. **D** Methylation levels of TUSC1 in normal gastric epithelial cells and tumor cells. **E**–**F** qRT-PCR and WB detection of TUSC1 expression in tumor cells following treatment with varying concentrations of 5-AZA-2. **G** Methylation levels of TUSC1 in tumor cells following treatment with varying concentrations of 5-AZA-2. **H**-**I** Assessment of viability and proliferation capacity of HGC-27 and NCI-N87 cells following treatment with 5-AZA-2. **J**-**K** Evaluation of the migration and invasion abilities of HGC-27 and NCI-N87 cells. **L**-**M** Detection of apoptosis and changes in the cell cycle of HGC-27 and NCI-N87 cells. **N** Examination of the expression levels of proliferation, apoptosis, and EMT marker proteins. *represents 0.01 ≤ *P* < 0.05, ** represents 0.001 ≤ *P* < 0.01, *** represents 0.0001 ≤ *P* < 0.001, **** represents *P* < 0.0001

To elucidate the underlying cause of TUSC1 downregulation in EJC, this study initially employed bioinformatics tools to predict the presence of highly methylated sites within the TUSC1 promoter region (Fig. 2A). Furthermore, a negative correlation between TUSC1 expression levels and the extent of methylation was observed (Fig. 2B). Subsequently, MSP assays were conducted to evaluate TUSC1 methylation levels in various samples, including 10 pairs of normal and patient tissues, as well as GES-1 and tumor cells. The results revealed that TUSC1 exhibited significantly lower methylation levels in adjacent tissues compared to EJC tissues, while HGC-27, AGS, and NCI-N87 cancer cells exhibited significantly higher methylation levels than GES-1 normal cells (Fig. 2C–D).

qRT-PCR and WB were employed to assess TUSC1 expression in HGC-27 and NCI-N87 cells following treatment with varying concentrations of the DNA methylation inhibitor, 5-AZA-2. Notably, TUSC1 expression increased with escalating concentrations of the demethylation agent (Fig. 2E–F). Subsequent MSP assays revealed that the methylation level of TUSC1 decreased with increasing concentrations of demethylating agents in HGC-27 and NCI-N87 cells (Fig. 2G). Moreover, CCK-8 and colony formation assays demonstrated that treatment with 5-AZA-2 (10 μM) effectively repressed the viability and proliferation of HGC-27 and NCI-N87 cells (Fig. 2H-2I).

Transwell assays were then conducted to evaluate the migration and invasion capabilities of HGC-27 and NCI-N87 cells before and after 5-AZA-2 (10 μM) treatment. The results indicated a significant reduction in cell migration and invasion following treatment, as compared to the control group (Fig. 2J-K). Additionally, flow cytometry analysis revealed that 5-AZA-2 (10 μM) treatment promoted apoptosis (Fig. 2L) and inhibited the cell cycle progression of HGC-27 and NCI-N87 cells (Fig. 2M). Furthermore, we assessed the proliferation, apoptosis, and expression of EMT marker proteins in 5-AZA-2-treated EJC cells through WB analysis, with results demonstrating significant reductions in the expression of Ki67 and vimentin, along with notable increases in caspase-3 and E-cadherin in HGC-27 and NCI-N87 cells treated with 5-AZA-2 (10 μM) (Fig. 2N). Collectively, these research findings provided compelling evidence of DNA methylation within the TUSC1 promoter region in EJC cells. Moreover, these methylation levels were found to intricately regulate TUSC1 expression.

### Regulatory effects of aberrant TUSC1 expression on the biological activities of EJC cells

**Fig. 3:**
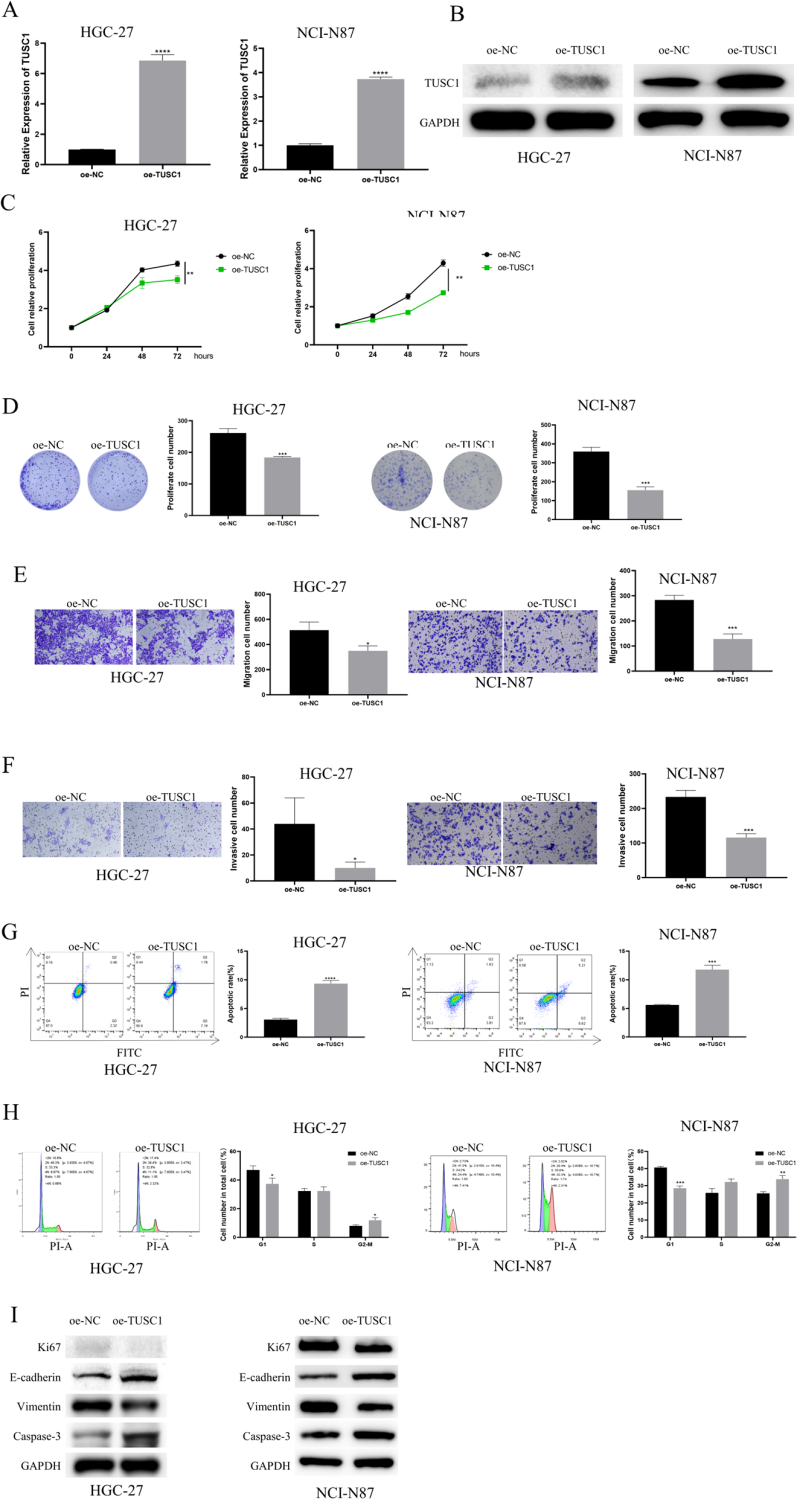
Regulatory effects of aberrant TUSC1 expression on the biological activities of EJC cells. **A**-**B** Measurement of TUSC1 mRNA and protein expression. **C** Assessment of cell viability. **D** Evaluation of HGC-27 cell colony formation capability. **E**–**F** Assessment of HGC-27 cell migration and invasion abilities. **G**–**H** Detection of apoptosis and cell cycle changes in HGC-27 cells. **I** Examination of the expression of proliferation, apoptosis, and EMT marker proteins in HGC-27 cells. *represents 0.01 ≤ *P* < 0.05, ** represents 0.001 ≤ *P* < 0.01, *** represents 0.0001 ≤ *P* < 0.001, **** represents *P* < 0.0001

To further investigate the role of TUSC1 in EJC, we established oe-NC and oe-TUSC1 human HGC-27 cell lines (with low TUSC1 expression in HGC-27 cells). qRT-PCR and WB results indicated that, compared to the control group, oe-TUSC1 significantly increased the mRNA and protein expression of TUSC1 in HGC-27 cells (Fig. 3A-B). Additionally, CCK-8 and colony formation assays showed that TUSC1 overexpression inhibited the viability and proliferation capacity of HGC-27 cells compared to the control group (Fig. 3C-D). Migration and invasion assays revealed that overexpressing TUSC1 in HGC-27 cells significantly reduced their migration and invasion abilities compared to the control group (Fig. 3E–F). Flow cytometry analysis demonstrated that TUSC1 overexpression promoted apoptosis and inhibited cell cycle progression in HGC-27 cells compared to the control group **(**Fig. 3G-H). Furthermore, WB analysis of the aforementioned cell groups illustrated that in TUSC1-overexpressing HGC-27 cells, the expression of Ki67 and vimentin decreased, while caspase-3 and E-cadherin increased (Fig. 3I). These results indicated that high TUSC1 expression suppressed the malignant progression of EJC cells.

### The impact of aberrant TUSC1 expression on in vivo EJC tumor growth and metastasis

**Fig. 4:**
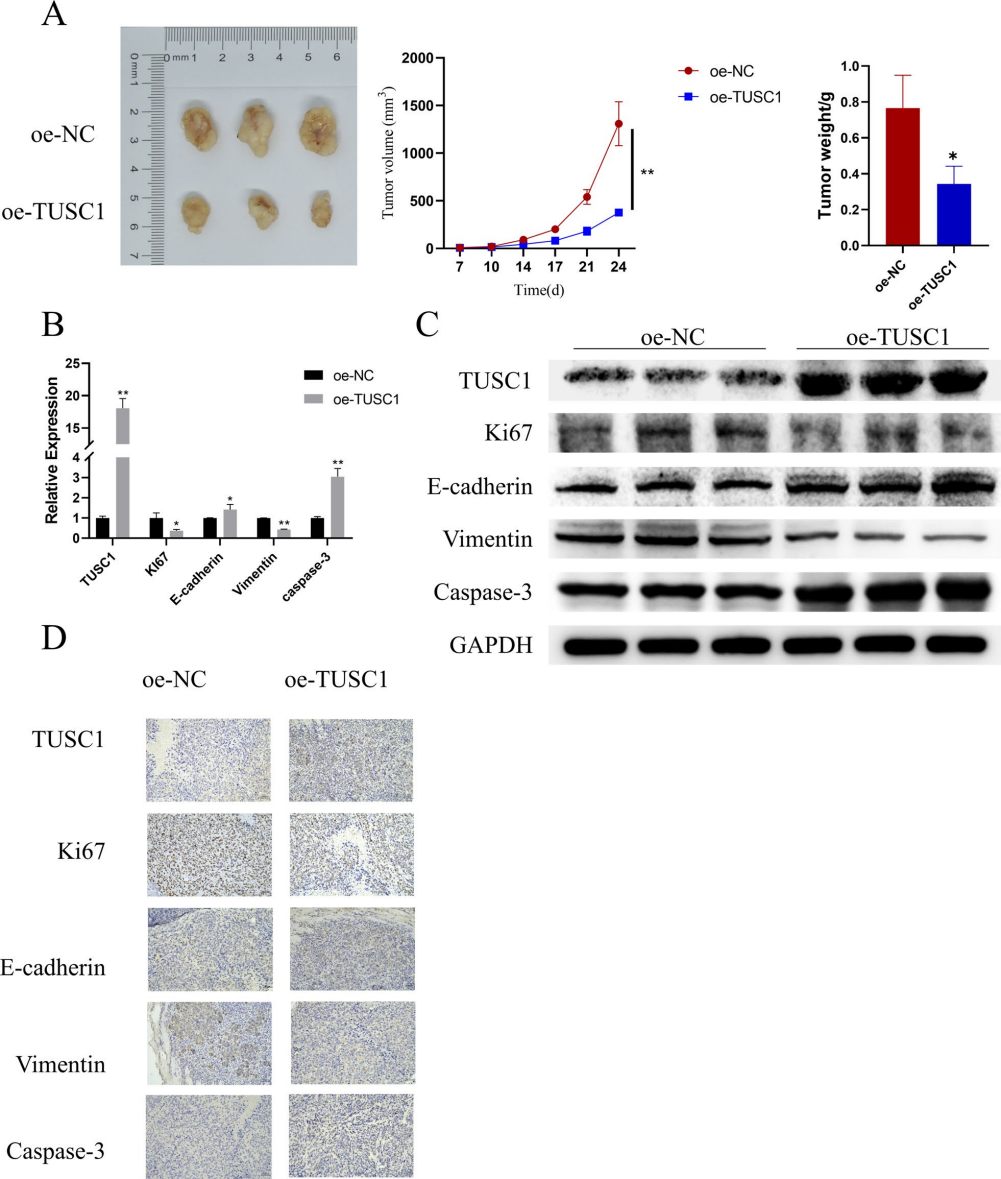
Effects of aberrant TUSC1 expression on EJC tumor growth and metastasis. **A** Assessment of EJC tumor size and weight. **B**–**D** qRT-PCR, WB, and IHC were used to detect the expressions of TUSC1, proliferation, EMT, and apoptosis marker proteins in tumor tissues of nude mice, respectively. *represents 0.01 ≤ *P* < 0.05, ** represents 0.001 ≤ *P* < 0.01

To delve into the influence of TUSC1 on tumor growth, we transfected HGC-27 cells with oe-NC/oe-TUSC1 and then subcutaneously implanted these cell lines into the right flank of nude mice, establishing a xenograft mouse model. Tumor size and volume were monitored, revealing that the oe-TUSC1 group exhibited reduced tumor weight and volume (Fig. 4A). Subsequently, we examined TUSC1 expression and the expression of proliferation, EMT, and apoptosis marker proteins in the tumor tissues of various groups using qRT-PCR, WB, and IHC, respectively. The results consistently demonstrated increased expression of TUSC1, caspase-3, and E-cadherin, along with decreased expression of Ki67 and vimentin in the oe-TUSC1 group (Fig. 4B–D). These research findings indicated that upregulation of TUSC1 in vivo can hinder EJC tumor growth and metastasis.

### Upregulation of TUSC1 promotes activation of the p53 signaling pathway

**Fig. 5:**
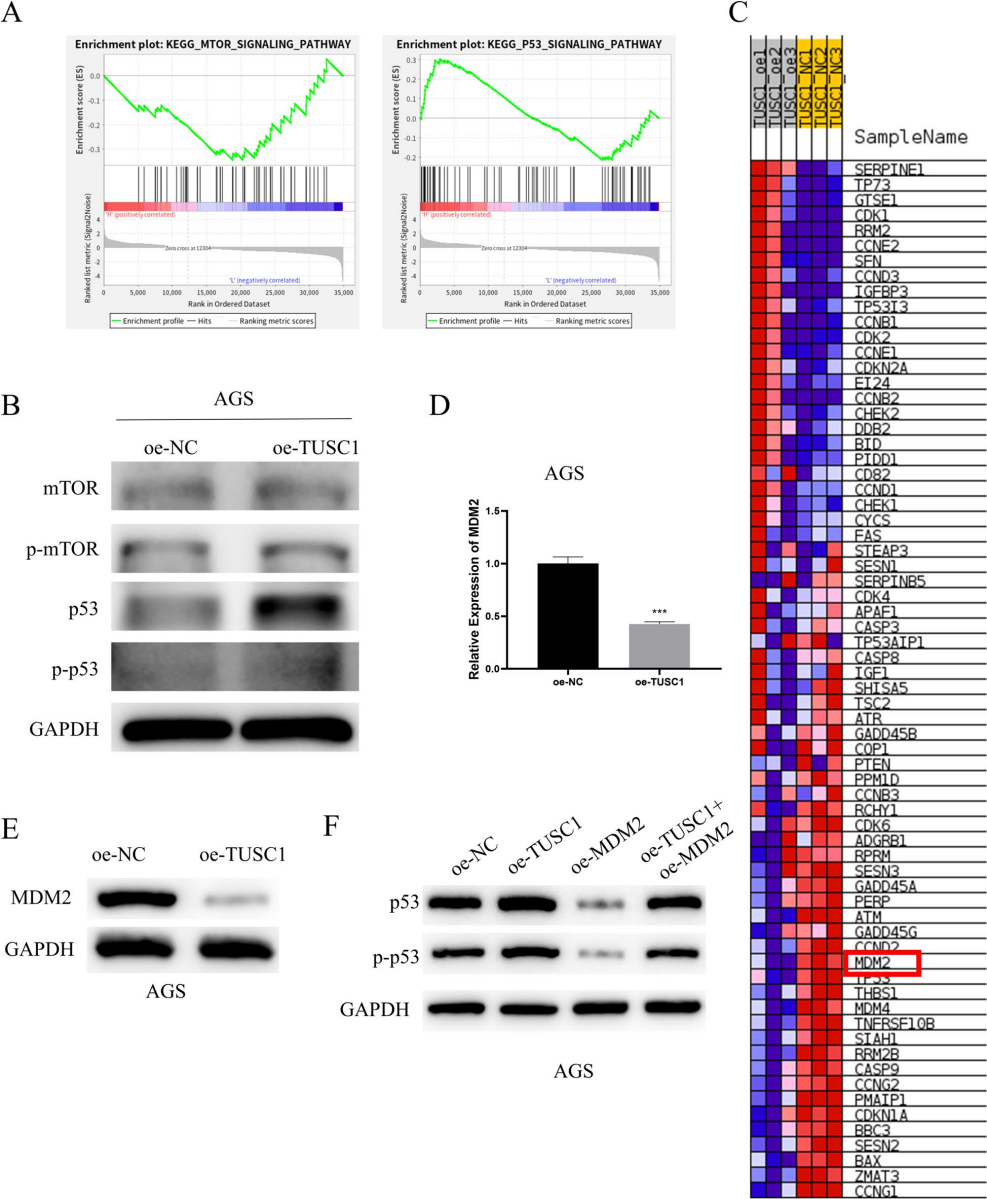
Effects of TUSC1 on mTOR and p53 signaling pathways. **A** Pathway analysis of TUSC1 enrichment. **B** The expression of proteins related to mTOR and p53 signaling pathways was detected. **C** GSEA results presented the genes associated with the p53 signaling pathway. (D-E) qRT-PCR and WB were used to detect the expressions of MDM2. (F) WB was used to detect the expressions of proteins related to p53 signaling pathways. *** represents 0.0001 ≤ *P* < 0.001

Ground on the results mentioned above, we observed that the high expression of TUSC1 suppressed the malignant biological behavior of EJC. Subsequently, we aimed to understand the downstream mechanisms influenced by TUSC1 in EJC. Initially, we performed transcriptome sequencing on EJC cells transfected with oe-NC and oe-TUSC1 (Attachment 1), followed by GSEA analysis, which indicated an enrichment of TUSC1 in the mTOR and p53 signaling pathways (Fig. 5A). Subsequently, by constructing oe-NC and oe-TUSC1 AGS cell lines, we examined the levels of mTOR and p53 pathway-related proteins in AGS cells using WB, with results displaying a notable increase in the expression of p-p53 following TUSC1 overexpression, with no significant effect on mTOR (Fig. 5B). p53 is a crucial tumor suppressor gene, and mutations in p53 often lead to the development of many malignant tumors [17]. Then, we identified the gene Mouse Double Minute 2 protein (MDM2), which is closely related to the p53 signaling pathway, based on the results of GSEA analysis (Fig. 5C). MDM2, the major negative regulator of p53, induces p53 degradation and inactivates its tumor-suppressing activity [18]. Thus, we hypothesized that TUSC1 affects the p53 pathway by regulating MDM2 expression. We detected MDM2 expression in AGS cells using qRT-PCR and WB and found that MDM2 expression was significantly reduced after overexpression of TUSC1 (Fig. 5D-E). Subsequently, we constructed AGS cell lines with oe-NC, oe-TUSC1, oe-MDM2, and oe-TUSC1 + oe-MDM2, and the WB results showed a significant increase in the expression of p-p53 after overexpression of TUSC1, a significant decrease in the expression of p-p53 after overexpression of MDM2, and no significant difference in the expression of p-p53 compared with the control cells after overexpression of both TUSC1 and MDM2 (Fig. 5F). Therefore, we hypothesized that TUSC1 may mediate the p53 pathway by negatively regulating the expression of MDM2, thereby affecting the development of EJC. These research findings suggested that the upregulation of TUSC1 promoted the stimulation of the p53 signaling pathway.

### TUSC1 suppresses the malignant progression of EJC cells via activation of the p53 signaling pathway

**Fig. 6:**
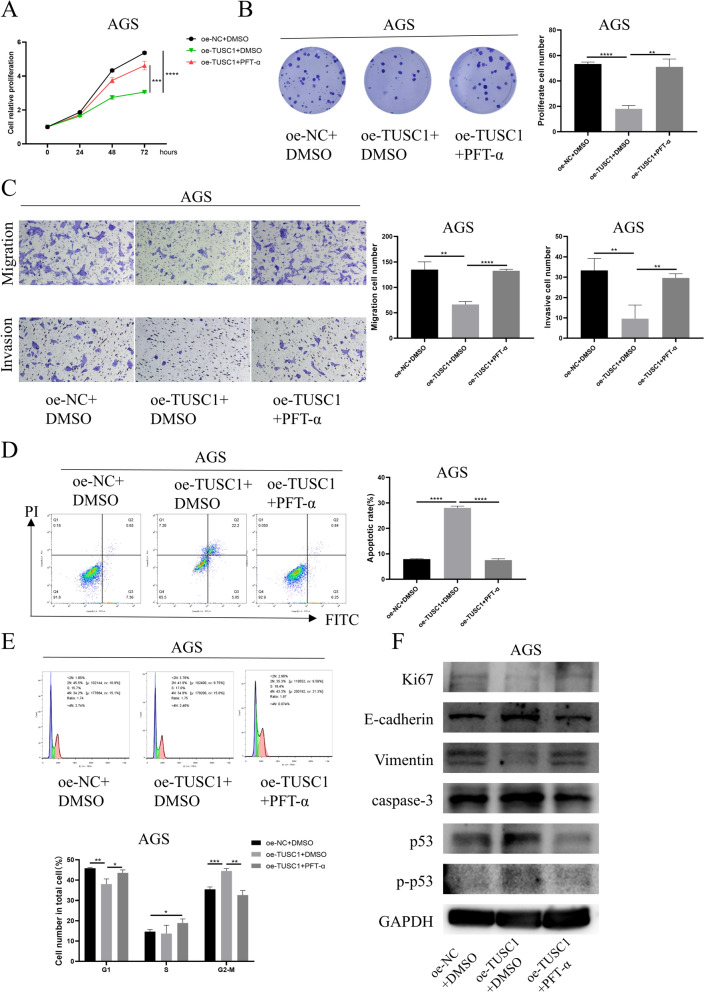
The Effect of TUSC1 on EJC Cell Progression through the p53 Signaling Pathway. **A** Assessment of cell viability. **B** Evaluation of colony formation capability in AGS cells. **C** Assessment of migration and invasion abilities in AGS cells. **D**–**E** Examination of apoptosis and cell cycle changes in AGS cells. **F** Evaluation of proliferation, apoptosis, EMT, and p53 signaling pathway-related protein expression in AGS cells. * represents 0.01 ≤ *P* < 0.05, ** represents 0.001 ≤ *P* < 0.01, *** represents 0.0001 ≤ *P* < 0.001, **** represents *P* < 0.0001

We conducted functional rescue experiments to further confirm that the upregulation of TUSC1 suppresses the malignant progression of EJC cells by activating the p53 signaling pathway. Initially, we established the following groups based on AGS cells: oe-NC + DMSO, oe-TUSC1 + DMSO, and oe-TUSC1 + PFT-α (p53 signaling pathway inhibitor). The results from CCK-8 and colony formation assays showed that compared to the control group, the viability and colony formation of AGS cells overexpressing TUSC1 were significantly reduced, but this effect was reversed when PFT-α was added (Fig. 6A-B). Migration and invasion assay results demonstrated that overexpression of TUSC1 tellingly inhibited the migration and invasion ability of AGS cells, which was attenuated when PFT-α was added, reducing the inhibitory effect of TUSC1 overexpression on cell migration and invasion (Fig. 6C). Flow cytometry analysis revealed that, compared to the control, TUSC1 overexpression promoted apoptosis and inhibited cell cycle progression in AGS cells. However, the addition of PFT-α weakened the impact of TUSC1 overexpression on AGS cell apoptosis and cell cycle progression (Fig. 6D-E). Furthermore, we assessed the expression of proliferation, apoptosis, and EMT marker proteins in AGS cells, along with the levels of p53 signaling pathway-related proteins, using WB. The results indicated that upregulation of TUSC1 significantly inhibited the expression of Ki67 and vimentin while promoting the expression of Caspase-3, E-cadherin, and p-p53 proteins. However, when TUSC1 overexpressing cells were treated with PFT-α, the expression of these proteins returned to levels similar to the control group (Fig. 6F). These findings suggested that TUSC1 suppressed the malignant progression of EJC cells via the stimulation of the p53 signaling pathway.

## Discussion

In recent years, there has been a significant global increase in the incidence of EJC [3], which exhibits more aggressive biological behavior than other types of GC. EJC is known to have a higher tendency for lymph node metastasis and generally carries poorer prognosis [3]. However, current research on GC primarily focuses on distal GC [19], and there is limited knowledge about the molecular genetics and pathogenic mechanisms that distinguish EJC from distal GC. As a result, the reasons for the rising incidence of EJC remain unclear. It is crucial to gain a deeper understanding of the mechanisms that influence the biological behavior of EJC to facilitate early diagnosis and comprehensive tumor management.

In this study, we initially reported the downregulation of TUSC1 in EJC and demonstrated that elevated TUSC1 expression affected tumor cell proliferation, migration, invasion, cell cycle, and apoptosis. Previous research has indicated that the TUSC1 gene is under-expressed in many human tumors such as gliomas and CRC [10, 20]. However, there is limited information available regarding its expression in EJC. In hepatocellular carcinoma (HCC), high methylation within the gene has been identified as a mechanism suppressing TUSC1 transcription. The hypermethylation of the TUSC1 gene has been proposed as a potential prognostic marker in HCC [11]. In our research endeavor, we have substantiated the existence of DNA methylation within the promoter region of TUSC1 in EJC cells, employing both bioinformatics analysis and molecular experimentation. This epigenetic modification manifests in the downregulation of the TUSC1 gene, thus aligning with and reinforcing the findings of prior scholarly investigations. Presently, DNA methylation is a hot topic in cancer research, including GC [21]. For instance, WD repeat protein 41 (WDR41) is under-expressed in triple-negative breast cancer, and methylated WDR41 positively regulates the AKT/GSK-3β/β-catenin pathway, promoting tumor proliferation, migration, and invasion [22]. Additionally, in GC, MUC6 expression is governed by promoter methylation, and the methylated state of the MUC6 promoter can significantly downregulate MUC6 in tumors, facilitating cancer progression [23]. Similarly, we found that the DNA methylation inhibitor 5-AZA-2 can reverse TUSC1 methylation, inhibit EJC cell proliferation, migration, and invasion, and enhance apoptosis rates. Collectively, these results underscore the critical role of DNA methylation regulation in tumor progression, and therapeutic strategies targeting DNA methylation may represent a novel direction for EJC treatment.

To further elucidate the potential molecular mechanisms by which TUSC1 influences EJC progression, we analyzed the impact of TUSC1 on downstream signaling pathways. Through transcriptome sequencing and GSEA analysis, we identified TUSC1 enrichment in the p53 signaling pathway. Subsequent cellular experiments confirmed that elevated TUSC1 expression promoted the expression of proteins related to the p53 signaling pathway. P53 is a crucial tumor suppressor gene, and its mutations are closely associated with tumorigenesis [24]. Most studies indicate that p53 inhibits tumor cell proliferation and promotes apoptosis [25, 26]. For instance, UPK3A is significantly upregulated in GC and fosters tumor proliferation, migration, and invasion by inactivating the p53 signaling pathway [27]. Lu et al. [28] found that the lncRNA SAMD12-AS1 can interact with DNMT1 to enhance its expression, thereby inhibiting the p53 signaling pathway and promoting GC progression.

Here, we further demonstrated that the inhibitory effects of high TUSC1 expression on the malignant progression of EJC cells can be attenuated by the addition of a p53 signaling pathway inhibitor. These suggested that targeting TUSC1 or activating the p53 pathway may serve as novel strategies for the prevention and treatment of EJC.

In summary, our results demonstrated that TUSC1 was downregulated in EJC, and we detected high methylation sites within the TUSC1 promoter region, where methylation levels influence TUSC1 expression. Upregulation of TUSC1 can promote the activation of the p53 signaling pathway and inhibit the proliferation and migration of EJC cells by mediating the p53 pathway. This study revealed a novel regulatory mechanism by which TUSC1 influenced EJC progression, providing new strategies for improving the treatment of EJC patients. However, our study has limitations. The small size of our patient cohort (n = 56) indicates the need for recruiting a larger population to strengthen our conclusions. Furthermore, further investigation into the mechanisms underlying the impact of TUSC1 on EJC is required. Nonetheless, our findings hold promise in shaping management and therapeutic strategies for EJC and offer fresh insights into the roles of TUSC1 and DNA methylation in the context of EJC.

### Supplementary Information

Additional file 1. 

Additional file 2. 

## Data Availability

The data and materials in the current study are available from the corresponding author on reasonable request.
